# Comparing the Impact of Pre-Operative Antibiotics on the Outcomes of Immediately Placed Dental Implants: A Retrospective Multi-Center Study

**DOI:** 10.3390/mps8040069

**Published:** 2025-07-01

**Authors:** Georgios S. Chatzopoulos, Larry F. Wolff

**Affiliations:** Department of Developmental and Surgical Sciences, Division of Periodontology, School of Dentistry, University of Minnesota, 515 Delaware Street SE, Minneapolis, MN 55455, USA; wolff001@umn.edu

**Keywords:** dental implant, osseointegration, immediate placement, survival analysis, treatment outcome

## Abstract

Background: This study aimed to evaluate and compare the survival rates of immediate dental implants (type 1) in patients who received different types of prophylactic antibiotics. Methods: This retrospective analysis examined data from 3351 immediate implants placed in 2391 patients (mean age 59.56 ± 13.42 years, 75.9% white, 53.6% female, 7.8% smokers, 6.7% with diabetes) within the BigMouth network between 2011 and 2022. Patient demographics, medical history, and the type of prophylactic antibiotic administered (amoxicillin, amoxicillin and clavulanic acid (Augmentin), clindamycin, azithromycin, ciprofloxacin, doxycycline, metronidazole) were analyzed in relation to implant survival or failure. Statistical analyses included descriptive statistics, chi-square tests, *t*-tests, Kaplan–Meier survival analysis, and Cox regression. All statistical analyses were performed with a significance level at *p* < 0.05. Results: The overall implant failure rate was 3.2% at the patient level (77 out of 2391 patients) and 1.9% at the implant level (65 out of 3351 implants), with a mean follow-up of 77 months. No significant associations were found between patient-related characteristics or implant position and implant failure, such as age (*p* = 0.84), gender (*p* = 0.30), or tobacco use (*p* = 0.83). Amoxicillin was the most frequently prescribed antibiotic (86.4%). Kaplan–Meier survival analysis revealed significantly shorter survival times for implants in patients who received ciprofloxacin and clindamycin compared to amoxicillin (n = 2894 implants) (*p* < 0.001). Cox regression analysis indicated a significantly increased risk of implant failure with ciprofloxacin (n = 5 implants) (HR: 16.50, *p* = 0.006) and clindamycin (n = 290 implants) (HR: 3.70, *p* < 0.001) compared to amoxicillin. Conclusion: The choice of prophylactic antibiotic significantly impacted the survival of immediate dental implants. Ciprofloxacin and clindamycin were associated with higher failure rates compared to amoxicillin. These findings underscore the importance of antibiotic selection in immediate implant procedures and highlight the need for further research to establish evidence-based guidelines for antibiotic prophylaxis in this context.

## 1. Introduction

Dental implants, pioneered by Branemark in the 1970s [1], represent a significant advancement in dentistry, offering a widely accepted method for replacing lost teeth. Generally, implants exhibit excellent long-term survival and success, with reported rates of 90–95% across a range of clinical scenarios and patient types [2,3,4,5,6]. Nevertheless, it is important to recognize that particular factors can make some individuals more susceptible to lower success rates and a higher risk of implant loss [7]. The conventional approach following tooth extraction required a 4- to 6-month period to allow for the healing of both the soft tissues and the underlying bone [8,9]. This waiting period was associated with several limitations, such as prolonged overall treatment time for implant placement, the inability to provide patients with a fixed temporary tooth, and considerable resorption of the alveolar ridge in both its soft tissue and bony components [10,11]. To overcome these challenges, a classification based on the timing of implant placement was introduced, defining four types: type 1 (immediate post-extraction), type 2 (4–8 weeks post-extraction), type 3 (12–16 weeks post-extraction), and type 4 (more than 4 months post-extraction) [12].

Immediate implant placement shows similar survival rates to other implant timing approaches [13,14] and offers several advantages. These include fewer surgical visits, good aesthetic outcomes, the potential for immediate temporary crowns, and a minimally invasive procedure [15]. Importantly, immediate implants help preserve the tissues around the implant, such as the natural contour of the gingival tissues and alveolus, thus minimizing bone resorption. This preservation is key to achieving superior aesthetics, characterized by an optimized emergence profile for the prosthetic crown and enhanced functional stability of the soft tissues surrounding the implant, ultimately leading to more optimal clinical and aesthetic results than with delayed implant placement [16]. Although immediate implant placement has potential advantages, it is also associated with several disadvantages and risks that can compromise treatment success. These include the higher chance of reduced primary stability of the implant, the potential need for supplementary surgical interventions, like grafting, and the technical complexity of the procedure itself [17,18,19,20].

The traditional approach in dental implant procedures involves prescribing preventive antibiotics due to the mouth’s bacterial environment and the risk of postoperative infections that could cause implant failure [21,22,23]. Consequently, antibiotics were considered crucial for managing these patients, serving both preventive and treatment roles [24,25]. However, a lack of standardized guidelines for antibiotic prescription in implant dentistry has led to significant variations in their use [25,26], and the potential for adverse effects and antibiotic resistance is a growing concern [27,28]. Interestingly, the scientific evidence supporting the routine use of systemic antibiotics in this field is not conclusive [29,30]. A European systematic review [31] revealed that most European professionals routinely prescribe antibiotics, often exceeding recommended guidelines, with amoxicillin being the most common and clindamycin used for penicillin allergies. Notably, a systematic review [32] indicated a strong association between clindamycin use and an increased risk of implant failure and infection. While the authors could not confirm penicillin allergy as an independent risk factor for early implant failure due to limitations in the data [32], the systematic review suggests potential alternatives to amoxicillin, such as azithromycin, clarithromycin, or metronidazole, especially as alternatives to clindamycin, until more definitive research is available [32].

The widespread use of antibiotics in implant dentistry contrasts with the absence of standardized pre-operative prescription guidelines. While numerous studies have explored various aspects of antibiotic use in implantology, existing evidence is still insufficient to establish definitive clinical protocols for antibiotic prophylaxis that directly compare the long-term effectiveness of different antibiotic regimens on the survival rates of immediate dental implants. This necessitates large-scale, long-term investigations to ascertain the true impact and optimize preventive antibiotic administration. We hypothesized that the type of prophylactic antibiotic administered prior to immediate dental implant placement significantly influences the survival rates of immediate dental implants. Therefore, this study aimed to evaluate and compare the survival rates of dental implants placed immediately following tooth extraction (type 1) in patients who received different types of antibiotics prophylactically prior to the surgical procedure. Understanding the impact of various antibiotic regimens on the success of immediate implants is crucial, especially considering the potential risks associated with this technique and the ongoing debate regarding antibiotic use in implant dentistry. A comprehensive review of electronic databases was conducted, which revealed a lack of studies specifically comparing the survival rates of immediate (type 1) dental implants in relation to different prophylactic antibiotic regimens. To the best of our knowledge, this study represents one of the first investigations specifically comparing the survival rates of dental implants placed immediately following tooth extraction (type 1) in relation to different prophylactic antibiotic regimens. This evidence gap, coupled with rising concerns about antibiotic resistance and potential adverse effects, makes large-scale investigations like ours essential for developing evidence-based clinical protocols.

## 2. Materials and Methods

### 2.1. Study Design

This retrospective analysis examined a cohort of patients who underwent dental implant treatment at university dental clinics participating in the BigMouth network, encompassing institutions including Harvard University and the Universities of Texas, California (San Francisco), Colorado, Loma Linda, Buffalo, Iowa, Minnesota, and Tufts. Data spanning from 2011 to 2022 were collected and analyzed, following ethical review and approval waivers from the University of Minnesota’s IRB (#STUDY00016865) and the BigMouth Consortium’s clinical review committee. The study adhered to the Declaration of Helsinki (2013 revision). The study’s reporting conforms to the STROBE guidelines.

BigMouth is a centralized oral health database that provides researchers with access to a vast collection of partially de-identified electronic health records. Hosted at UTHealth Houston and available through the open-source i2b2 platform, it has grown significantly since 2012, now encompassing over 7 million patient records contributed by at least 17 U.S. dental institutions. Its robust governance framework ensures data privacy, allows contributing institutions to maintain control, and provides standardized access for research and quality improvement projects.

### 2.2. Study Population

Patient records within the BigMouth network were screened to identify individuals who received dental implants between 2011 and 2022 based on completed oral evaluation codes (CDT D0150, D0120, D0180) and at least one implant placement (CDT D6010). Implant failure was identified using the removal code (CDT D6100). Critically, the study focused on patients who received antibiotic medication prophylactically prior to their implant surgery, including amoxicillin, amoxicillin and clavulanic acid (Augmentin), clindamycin, azithromycin, ciprofloxacin, doxycycline, and metronidazole, particularly those who underwent immediate implant placement (type 1). “Type 1” or “immediate” implant placement refers to the insertion of the dental implant into the fresh extraction socket during the same surgical appointment as the tooth extraction. Records lacking information on antibiotic administration were excluded.

### 2.3. Data Collection

Collected patient data included age, gender, ethnicity, race, tobacco use, and systemic medical conditions (cardiovascular, endocrine, infectious, kidney, musculoskeletal, neurological, and respiratory disorders). These factors, along with the type of prophylactic antibiotic administered, were analyzed for their potential influence on implant outcomes. The primary outcome was implant survival using a binary classification of survival or failure, with failure defined as implant removal due to lack of osseointegration (mobility). Time to failure was recorded from surgery to failure detection, with the last follow-up date used for surviving implants.

### 2.4. Statistical Analysis

Statistical analysis involved descriptive statistics, chi-square tests, and *t*-tests for demographic comparisons. Significant variables (*p* < 0.05) were further analyzed using multivariate logistic regression to calculate odds ratios. Implant survival at the implant level was assessed using Kaplan–Meier plots, and hazard ratios were calculated using the Cox regression model. Data analysis was performed using SPSS version 29.0, with a significance level of 0.05. The findings of this study aim to provide insights into the survival rates of dental implants placed immediately following tooth extraction (type 1) in relation to different prophylactic antibiotic regimens. By analyzing a large, multi-center patient cohort, this research seeks to contribute to evidence-based guidelines for antibiotic use in immediate implant procedures, considering patient demographics, medical history, and the specific antibiotics administered.

## 3. Results

The initial screening of 50,333 dental implants placed in 20,842 patients yielded a final cohort of 3351 immediate implants in 2391 individuals meeting the inclusion criteria. The patient-related characteristics of the total included population, as well as the patient groups with implant survival and failure, are shown in Table 1. The mean age of the cohort was 59.56 (SD: 13.42) years. The demographic profile indicated that 91.7% of patients belonged to non-Hispanic ethnic groups, 75.9% were of White/Caucasian race, 53.6% were female, 7.8% were smokers, and 6.7% had diabetes. The statistical analysis revealed no significant differences between the survival and failure groups for any of the patient-related characteristics examined (*p* > 0.05 for all variables). This suggests that factors like age, gender, ethnicity, race, tobacco use, and the presence of various systemic medical conditions (including hypertension, diabetes, and others) were not significantly associated with the likelihood of implant failure in this cohort. The overall implant failure rate in this study was 3.2% at the patient level (77 of 2391 patients), with 96.8% of patients (2314) having surviving implants. At the implant level, the failure rate was 1.9% (65 of 3351 implants).

A total of 3351 records of immediate implants were included in the present investigation, which were followed up for a mean 77 months (standard deviation: 49.72). The majority of the implants were placed in the maxilla (62.3%, n = 2088), with the remainder in the mandible (37.7%, n = 1263). The placement of implants was evenly divided between the anterior and posterior regions, with each accounting for 50% of the total (n ≈ 1676). Neither the implant region (*p* = 0.90) nor the jaw location (*p* = 0.70) had a significant effect on implant treatment outcome. The type of antibiotics given to those who exhibited implant failure and survival, as well as the total population, is shown in Table 2. The data reveal that amoxicillin was the most frequently prescribed antibiotic (86.4% of the total immediate implants), followed by clindamycin (8.7%). Notably, the implant failure group (1.9% of the total) exhibited a different distribution of antibiotic usage compared to the implant survival group (98.1%). A chi-square test confirmed that these differences in antibiotic type distribution between the two groups were statistically significant (*p* < 0.001).

**Figure 1:**
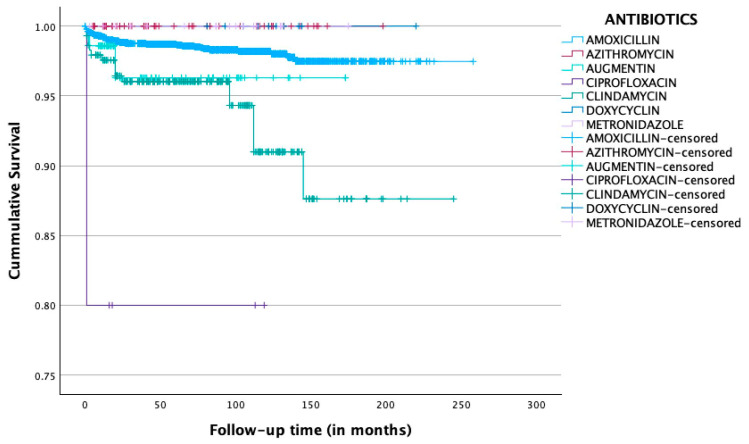
Kaplan–Meier curves comparing the cumulative survival of implants in patients who received one of the following pre-operative antibiotics: amoxicillin, azithromycin, amoxicillin and clavulanic acid (Augmentin), ciprofloxacin, clindamycin, doxycycline, and metronidazole.

Figure 1 shows Kaplan–Meier curves comparing the cumulative survival of implants in patients who received one of the following pre-operative antibiotics: amoxicillin, azithromycin, amoxicillin and clavulanic acid (Augmentin), ciprofloxacin, clindamycin, doxycycline, and metronidazole. The implant survival rates were as follows: 98.4% for amoxicillin, 100.0% for azithromycin, 97.2% for amoxicillin and clavulanic acid (Augmentin), 80.0% for ciprofloxacin, 94.1% for clindamycin, 100.0% for doxycycline, and 100.0% for metronidazole. The mean cumulative survival times for implants were 253.00 months (91% CI: 251.39–254.60) for amoxicillin, 167.10 months (95% CI: 158.91–175.28) for amoxicillin and clavulanic acid (Augmentin), 95.40 months (95% CI: 54.03–136.78) for ciprofloxacin, and 225.34 months (95% CI: 215.23–235.46) for clindamycin. A statistically significant difference was observed in the cumulative survival times between the various antibiotics (*p* < 0.001). Survival analysis demonstrated that immediate implants placed in patients premedicated with ciprofloxacin exhibited a significantly shorter mean cumulative survival time compared to those who received amoxicillin (*p* < 0.001), Augmentin (*p* = 0.033), azithromycin (*p* < 0.001), and metronidazole (*p* = 0.05). Similarly, the cumulative survival time was significantly shorter in the clindamycin group compared to the amoxicillin group (*p* < 0.001). Cox regression analysis revealed that implants in the ciprofloxacin group showed a statistically significant increased risk of failure compared to amoxicillin (HR: 16.50, 95% CI: 2.27–119.98, *p* = 0.006). In addition, implants in the clindamycin group demonstrated a significantly higher risk of failure (HR: 3.70, 95% CI: 2.18–6.46, *p* < 0.001) compared to the amoxicillin group.

## 4. Discussion

Using the BigMouth Dental Data Repository, this large retrospective cohort study compared the impact of various pre-operative antibiotics on the treatment outcome of implants inserted immediately following tooth extraction. The analysis included 3351 implants in 2391 individuals followed for a mean of 77 ± 49.72 months (range: 0–258 months). The primary findings revealed the following.

In this study, the overall implant failure rate was 3.2% at the patient level, meaning 77 out of 2391 patients experienced implant failure, while 96.8% (2314 patients) had surviving implants. At the implant level, the failure rate was 1.9%, with 65 out of 3351 implants failing. No significant associations were found between implant failure and patient-related characteristics, such as age, gender, ethnicity, race, tobacco use, systemic conditions, or implant position (region and jaw). Amoxicillin was the most commonly prescribed antibiotic, accounting for 86.4% of cases, followed by clindamycin at 8.7%. However, implant survival rates differed among antibiotic groups, with amoxicillin showing a 98.4% survival rate and ciprofloxacin showing 80.0%. Specifically, ciprofloxacin was linked to a significantly shorter mean cumulative survival time when compared to amoxicillin, Augmentin, azithromycin, and metronidazole. Similarly, clindamycin was associated with a significantly shorter mean cumulative survival time compared to amoxicillin. Cox regression analysis further indicated a significantly increased risk of implant failure with ciprofloxacin (HR: 16.50) and clindamycin (HR: 3.70) when compared to amoxicillin.

The optimal timing for dental implant placement, whether immediately following tooth extraction or delayed to allow for tissue healing, is still debated in implant dentistry. A systematic review of immediate implants encompassing 46 prospective studies (including 10 randomized controlled trials) and data from 2098 implants with a mean 2-year follow-up reported an estimated 2-year survival rate of 98.4% [33]. The authors emphasized the influence of antibiotic regimens on implant success, highlighting the importance of post-surgical antibiotics. Specifically, the annual failure rate was higher (1.78%) with only a single pre-operative antibiotic dose compared to a 5–7-day postoperative course (0.51%) or a combination of single pre-operative and 5–7-day postoperative doses (0.75%), regardless of the antibiotic used. Studies on risk factors for immediate implant failure have shown inconsistent results, but one study found that patients not receiving amoxicillin had a 3.34 times higher risk of implant failure [34].

Ciprofloxacin exhibited a significantly shorter mean cumulative survival time compared to those who received amoxicillin (*p* < 0.001), Augmentin (*p* = 0.033), azithromycin (*p* < 0.001), and metronidazole (*p* = 0.05). Implants in the ciprofloxacin group showed a statistically significant increased risk of failure compared to amoxicillin (HR: 16.50, *p* = 0.006). Current evidence on how different antibiotics affect implant treatment outcomes remains inconclusive. Wagenberg et al. [35] observed slightly less crestal bone loss with penicillin compared to other antibiotics, although this difference was not statistically significant. Notably, the specific antibiotics used in that study were not specified. In their 2020 study, researchers found no significant differences in bone loss at maxillary or mandibular sites when comparing amoxicillin and azithromycin regimens in patients with self-reported penicillin allergies [36]. Ciprofloxacin may hinder bone healing by negatively affecting osteoblast growth. Research on human bone cells indicates that typical local infection doses of ciprofloxacin can inhibit the proliferation of osteoblast-like cells [37]. This inhibition can lead to delayed bone healing and reduced bone density, potentially interfering with osseointegration. A study in rats with femoral fractures showed that ciprofloxacin delayed fracture healing compared to controls (cefazolin or no treatment), resulting in decreased bone strength and stiffness [38]. Histological analysis in that study also revealed abnormal cartilage and bone formation in the ciprofloxacin group. The study concluded that ciprofloxacin may impair early-stage fracture healing [38]. Another rat study comparing various fluoroquinolones (norfloxacin, ofloxacin, pefloxacin, ciprofloxacin) found that fracture healing was better in the control group (no antibiotics) than in any antibiotic group [39]. Therefore, the reduced implant survival time in patients premedicated with ciprofloxacin may be linked to the drug’s negative impact on bone healing.

Similarly, the cumulative survival time was significantly shorter in the clindamycin group compared to the amoxicillin group (*p* < 0.001). Implants in the clindamycin group demonstrated a significantly higher risk of failure (HR: 3.70, *p* < 0.001) compared to the amoxicillin group. The connection between self-reported penicillin allergies and dental implant failure was examined in a retrospective study from the New York University College of Dentistry [40]. The study revealed a notably higher implant failure rate (17.1%) in patients reporting a penicillin allergy compared to non-allergic patients treated with amoxicillin (8.4%). Notably, within the penicillin-allergic group, clindamycin use was associated with a higher failure rate than amoxicillin or alternative antibiotics. The researchers concluded that amoxicillin use was linked to reduced implant failure rates, while patients with self-reported penicillin allergies faced a significantly elevated risk of early implant failure [40]. Consistent with these findings, a systematic review highlighted a strong association between clindamycin use and an increased risk of implant failure, as well as a potential six-fold increase in the risk of infection [32]. A further systematic review and meta-analysis, encompassing four studies, determined that patients treated with clindamycin were over three times more likely to experience implant failure [41]. However, due to limitations in the current evidence, it remains unclear whether the allergy itself, clindamycin use, or a combination of both factors contributes to this increased risk. Our finding of a significantly higher risk of implant failure associated with clindamycin use aligns with previous research demonstrating a strong link between clindamycin and increased implant failure rates.

This retrospective study, while providing valuable insights into antibiotic prophylaxis and immediate implant outcomes, has inherent limitations. The retrospective design relies on existing records, which may suffer from incompleteness or inaccuracies, and this limits control over data collection. Data on specific procedural variables, such as implant manufacturer/type, immediate vs. delayed loading protocols, the use of bone grafting materials, or the experience level of the individual surgeons, were not uniformly available within the de-identified electronic health records of the BigMouth network. Moreover, a further limitation stemming from the study’s retrospective design is that granular data on antibiotic administration, such as the precise dosage, the route, the full duration of the prescribed regimen, and the reason for using a specific antibiotic regimen (possible allergy), were not consistently available within the electronic health records and therefore could not be analyzed. While the overall sample size was large, the study was not specifically powered a priori to detect significant differences within smaller racial or ethnic subgroups, and the number of failures within these subgroups was low. The study’s multi-center nature, while a strength in generalizability, also introduces potential heterogeneity in treatment protocols and data recording. Furthermore, while patient-related factors were analyzed, the possibility of unmeasured confounders influencing implant outcomes cannot be excluded. Finally, the variability in antibiotic prescription practices, a problem the study itself highlights, remains a limitation, as it introduces complexity in interpreting the impact of specific antibiotics.

A significant limitation of this study, particularly in the analysis of antibiotic associations with implant failure, is the substantial discrepancy in sample sizes between the antibiotic exposure groups. For instance, the number of implants associated with amoxicillin use (n = 2894) was considerably larger than those associated with clindamycin use (n = 290). This pronounced imbalance in group sizes inherently limits the statistical power to detect significant differences within the smaller group and necessitates extreme caution in interpreting comparative findings. Another limitation of our findings concerning antibiotic associations with implant failure pertains to the extremely small sample size for certain antibiotic exposures. Notably, the analysis for ciprofloxacin was based on data from only five implants. While a high hazard ratio (HR: 16.50) was observed in this subsample, it is imperative to interpret this finding with extreme caution. The statistical power to detect meaningful or reliable associations from such a limited number of observations is severely compromised. Consequently, this result should be considered hypothesis-generating at best and not as a definitive conclusion regarding the relationship between ciprofloxacin use and implant failure. This small sample size makes the estimate highly susceptible to random variability and potential outliers, thus limiting its generalizability.

However, the study also possesses several notable strengths. The large sample size significantly enhances the statistical power of the analysis, and the multi-center design increases the generalizability of the findings. The long mean follow-up period of 77 months provides valuable long-term data on implant survival. Moreover, the study’s use of the BigMouth Dental Data Repository facilitated the efficient analysis of a substantial dataset. Importantly, this research directly addresses a critical knowledge gap by specifically investigating the impact of different prophylactic antibiotic regimens on immediate implant outcomes, an area where standardized guidelines are lacking.

Future research should prioritize addressing the identified limitations and building on the study’s strengths. Randomized controlled trials are essential to establish causal relationships between antibiotic protocols and implant success. Developing standardized antibiotic protocols for immediate implant placement is crucial to reduce variability and improve clinical consistency. Further research could explore the pharmacokinetic and pharmacodynamic profiles of antibiotics in this context, delve into the role of the oral microbiome, and conduct cost-effectiveness analyses of different antibiotic strategies. Ultimately, continued long-term studies and investigations into antibiotic resistance are necessary to refine clinical practice and optimize patient care in immediate implant dentistry.

## 5. Conclusions

This retrospective study analyzed 3351 immediate implants in 2391 patients over a mean follow-up of 77 months, revealing an overall implant failure rate of 3.2% at the patient level and 1.9% at the implant level. While patient-related factors, such as age, gender, ethnicity, race, tobacco use, and systemic conditions, did not show a statistically significant influence on implant failure, the type of prophylactic antibiotic administered did play a significant role. Amoxicillin was the most frequently prescribed antibiotic, accounting for 86.4% of cases. The distribution of antibiotic usage varied significantly between implant survival and failure groups.

Notably, implants in patients premedicated with clindamycin and ciprofloxacin demonstrated significantly shorter survival times and a higher risk of failure compared to those receiving amoxicillin. However, it is crucial to interpret these associations with appropriate caution. The comparison of antibiotic groups was limited by unbalanced sample sizes, particularly between amoxicillin (n = 2894) and clindamycin (n = 290). More critically, the observed association with ciprofloxacin stemmed from an extremely small sample size of only five implants. Due to this severe limitation, the high hazard ratio observed for ciprofloxacin should be considered hypothesis-generating at best, and no definitive conclusions can be drawn regarding its impact on implant failure from our data.

These findings underscore the potential importance of antibiotic selection in immediate implant procedures. However, given the identified methodological limitations, particularly concerning sample sizes for less commonly prescribed antibiotics, further research is essential. Future studies with larger, more balanced cohorts are needed to establish standardized, evidence-based guidelines for antibiotic prophylaxis in immediate implant procedures.

## Figures and Tables

**Table 1:** Patient-related characteristics of the total included population as well as the patient groups with implant survival and failure.

Patient-Related Characteristics		Total n = 2391 (100%)	Implant Survival n = 2314 (96.8%)	Implant Failure n = 77 (3.2%)	*p*-Value
Age (mean (SD))		59.56 (13.42)	59.57 (13.48)	59.25 (11.74)	0.84
Gender (%)	Female	1281 (53.6)	1235 (53.4)	46 (59.7)	0.30
	Male	1110 (46.4)	1079 (46.6)	31 (40.3)	
Ethnicity (%)	Non-Hispanic	2193 (91.7)	2119 (91.6)	74 (96.1)	0.22
	Hispanic	117 (4.9)	114 (4.9)	3 (3.9)	
	Others	81 (3.4)	81 (3.5)	0 (0.0)	
Race (%)	White	1814 (75.9)	1752 (75.7)	62 (80.5)	0.26
	Asian	113 (4.7)	111 (4.8)	2 (2.6)	
	African American	87 (3.6)	81 (3.5)	6 (7.8)	
	Hispanic or Latino	147 (6.1)	143 (6.2)	4 (5.2)	
	Pacific Islander	6 (0.3)	6 (0.3)	0 (0.0)	
	American Indian or Alaskan Native	6 (0.3)	6 (0.3)	0 (0.0)	
	Others	218 (9.1)	215 (9.3)	3 (3.9)	
Tobacco use (%)		187 (7.8)	182 (7.9)	5 (6.5)	0.83
Hypertension (%)		385 (16.1)	371 (16.0)	14 (18.2)	0.64
Marijuana use (%)		25 (1.0)	23 (1.0)	2 (2.6)	0.19
Diabetes (%)		161 (6.7)	155 (6.7)	6 (7.8)	0.64
Thyroid disorder (%)		157 (6.6)	149 (6.4)	8 (10.4)	0.16
HIV (%)		5 (0.2)	5 (0.2)	0 (0.0)	1.00
Kidney disease (%)		107 (4.5)	101 (4.4)	6 (7.8)	0.16
Arthritis (%)		267 (11.2)	259 (11.2)	8 (10.4)	1.00
Osteoporosis (%)		79 (3.3)	74 (3.2)	5 (6.5)	0.11
Depression (%)		149 (6.2)	143 (6.2)	6 (7.8)	0.48
Seizures/epilepsy (%)		7 (0.3)	7 (0.3)	0 (0.0)	1.00
Asthma (%)		142 (5.9)	138 (6.0)	4 (5.2)	1.00
Sleep apnea (%)		55 (2.3)	52 (2.2)	3 (3.9)	0.26

Abbreviations: SD, standard deviation.

**Table 2:** Type of antibiotics given to those who exhibited implant failure and survival as well as the total population.

Antibiotics	Total n = 3351 (100%)	Implant Survival n = 3286 (98.1%)	Implant Failure n = 65 (1.9%)
Amoxicillin (%)	2894 (86.4)	2849 (98.4)	45 (1.6)
Azithromycin (%)	61 (1.8)	61 (100.0)	0 (0.0)
Amoxicillin and clavulanic acid (Augmentin) (%)	71 (2.1)	69 (97.2)	2 (2.8)
Ciprofloxacin (%)	5 (0.1)	4 (80.0)	1 (20.0)
Clindamycin (%)	290 (8.7)	273 (94.1)	17 (5.9)
Doxycycline (%)	11 (0.3)	11 (100.0)	0 (0.0)
Metronidazole (%)	19 (0.6)	19 (100.0)	0 (0.0)

## Data Availability

Data available upon request from the corresponding author.
